# The Overlap Syndrome of Obstructive Sleep Apnea and Chronic Obstructive Pulmonary Disease: A Systematic Review

**DOI:** 10.3390/biomedicines11010016

**Published:** 2022-12-21

**Authors:** Katarzyna Czerwaty, Karolina Dżaman, Krystyna Maria Sobczyk, Katarzyna Irmina Sikorska

**Affiliations:** Department of Otolaryngology, Centre of Postgraduate Medical Education, Marymoncka 99/103, 01-813 Warsaw, Poland

**Keywords:** obstructive sleep apnea (OSA), chronic obstructive pulmonary disease (COPD), overlap syndrome, polysomnography, spirometry

## Abstract

Chronic obstructive pulmonary disease (COPD) and obstructive sleep apnea (OSA) are common diseases that strongly impact the quality and length of life. Their coexistence is determined by overlap syndrome (OS). This systematic review aims to define the significance of these comorbidities according to the current state of knowledge. For this systematic review, we searched PubMed, Scopus, and Cochrane for studies published between 2018 and 26 October 2022, to find original, observational, human studies published in English, where the diagnosis of COPD was according to the Global Initiative for Obstructive Lung Disease guidelines and the diagnosis of OSA was based on polysomnography. The quality of studies was assessed using the Newcastle–Ottawa quality assessment tool for cohort and case–control studies, as well as its modification for cross-sectional studies. Of the 1548 records identified, 38 were eligible and included in this systematic review. The included studies covered a total population of 27,064 participants. This paper summarizes the most important, up-to-date information regarding OS, including the prevalence, meaning of age/gender/body mass index, polysomnography findings, pulmonary function, comorbidities, predicting OSA among COPD patients, and treatment of this syndrome.

## 1. Introduction

Chronic obstructive pulmonary disease (COPD) and obstructive sleep apnea (OSA) are common disorders with substantial impacts on global health, causing a significant economic burden, and they often co-occur with one another [1].

OSA is characterized by a complete cessation (apnea) or significant decrease (hypopnea) in airflow during sleep, caused by recurrent episodes of upper-airway collapse, leading to nocturnal oxyhemoglobin desaturations and arousals from rest [2]. The recurrent arousals that occur in OSA lead to neurocognitive consequences, daytime sleepiness, and reduced quality of life (QoL). Because of apneas and hypopneas, patients experience hypoxemia and hypercapnia, which result in increasing levels of catecholamine, oxidative stress, and low-grade inflammation that lead to the appearance of cardiometabolic consequences of OSA [3].

COPD is a chronic inflammatory lung disease defined by persistent, usually progressive airflow limitation (AFL) [4]. Changes in lung mechanics lead to the main clinical manifestations of dyspnea, cough, and chronic expectoration [5,6]. Furthermore, patients with COPD often suffer from anxiety and depression [7], and their risk of OSA and insomnia is higher than that of those hospitalized for other reasons [8]. Although COPD is twice as rare as asthma, it is the cause of death eight times more often [9].

The association of COPD and OSA is known as the COPD–OSA “overlap” syndrome (OS) [10] and is linked with a poor prognosis. Although COPD and OSA are both highly prevalent diseases, it is unclear whether each disorder predisposes patients to a higher incidence of the other [11]; however, they can influence one another in terms of pathophysiology [1]. Both conditions are characterized by severe clinical symptoms and are associated with significant morbidity and mortality [12,13], especially when they co-occur [14]. Usually, primary care physicians, pulmonologists, or sleep specialists make a single diagnosis of either OSA or COPD. COPD diagnosis is simple and inexpensive, whereas OSA diagnosis requires overnight polysomnography (PSG)—a time-consuming and expensive test of limited availability [15,16].

This systematic review aims to provide the essential findings in the field of OS, including prevalence, possible predictors, association with clinical outcomes, and severity compared to both COPD and OSA patients.

## 2. Materials and Methods

### 2.1. Literature Search

**Figure 1:**
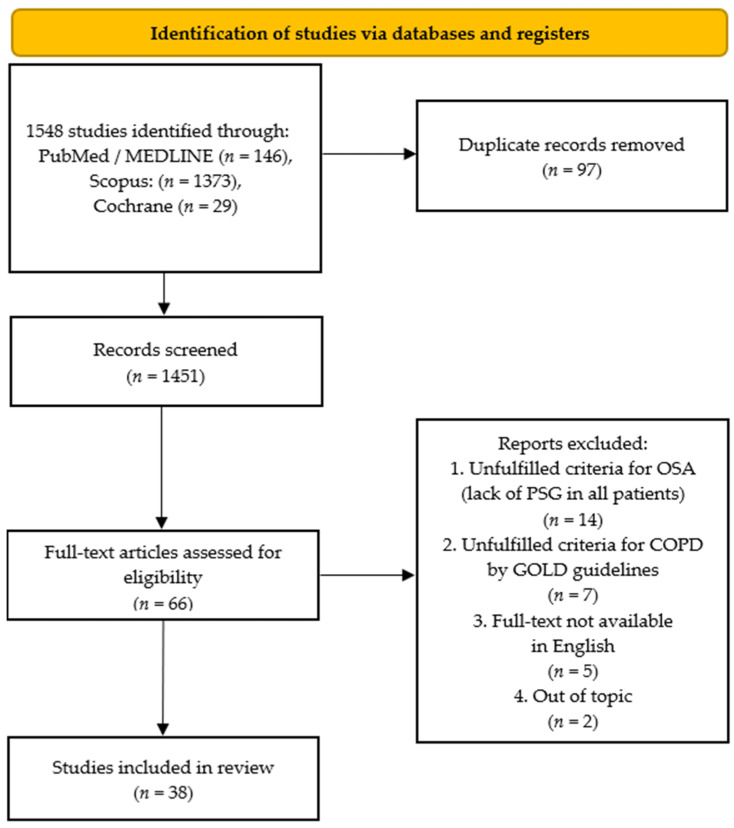
The Preferred Reporting Items for Systematic Reviews and Meta-Analyses (PRISMA) flow diagram of the systematic literature search, showing the study selection process.

This systematic review was performed following the Preferred Reporting Items for Systematic Reviews and Meta-Analyses (PRISMA; Berlin, Germany) guidelines [17]. A flowchart is provided in Figure 1.

A systematic literature review was carried out with the use of three databases: PubMed/MEDLINE, Scopus, and Cochrane. Studies providing any association between COPD and OSA were searched. The following keywords or combinations were used to retrieve the studies: “chronic obstructive pulmonary disease”, “chronic obstructive lung disease”, “chronic obstructive airway disease”, “COPD” or “COAD” merging “obstructive sleep apnea”, “sleep apnea syndrome”, or “OSA”. The detailed search strategy is presented in Table S1. The last search was run on 26 October 2022 on each database. It was not necessary to contact the authors of the retrieved research articles for additional information. The methods of the analysis and inclusion criteria were specified in advance. The inclusion and exclusion criteria for the retrieved studies are presented in Table 1.

Duplicates were discarded using the automatic search function in EndNote 20 (Clarivate Analytics, London, UK), followed by a manual search. The titles and abstracts of the remaining manuscripts were screened independently by two reviewers (K.C. and K.M.S) according to the inclusion and exclusion criteria (Table 1). The diagnosis of COPD was based on the spirometric criterion for AFL—a post-bronchodilator fixed ratio of forced expiratory volume in 1 second/forced vital capacity (FEV_1_/FVC) less than 0.70, according to the Global Initiative for Obstructive Lung Disease (GOLD) classification for COPD [4]. OSA diagnosis was made based on a PSG examination, which is still the gold-standard diagnostic tool [16].

In cases of disagreement regarding the eligibility of a study, a third investigator (K.D.) was involved for consensus to be reached. Subsequently, two of this study’s authors (K.C. and K.M.S) independently screened the full-text articles for inclusion. For the second time, in case of disagreement, a consensus was reached on inclusion or exclusion by the third investigator (K.D.). The details of the selection process are presented in a customized PRISMA flow diagram (Figure 1). We selected original studies concerning the OS, and the results of the retrieved articles are summarized and discussed in this review.

### 2.2. Data Extraction

A customized data extraction sheet was subsequently used for the collection of the following information: first author’s name, year and country of publication, study design, study aim(s), study population, and main results. One of the review authors extracted the abovementioned data from eligible studies, while the second author double-checked their correctness.

### 2.3. Risk-of-Bias Assessment

The risk of bias was assessed by two independent reviewers (K.C. and K.I.S.) using the Newcastle–Ottawa quality assessment scale for cohort and case–control studies [18] and its modified version adapted for cross-sectional studies [19]. Any discrepancies in judgments regarding the risk of bias were resolved by the third author (K.D.). A total score of 0–3 was considered unsatisfactory, 4–5 points was satisfactory, 6–7 points was good, and 8–9 points was very good. Additionally, other potential sources of bias (not included in the scale) are described in the Discussion section.

## 3. Results

### 3.1. Search Results

Details of the selection process are summarized in a PRISMA flowchart in Figure 1. The process of systematically searching the literature yielded 1548 citations from PubMed, Scopus, and Cochrane. Of these, 85 duplicates found automatically and 12 additional duplicates found manually were removed. Based on reading of the titles and abstracts, 1385 items were eliminated as not meeting the inclusion criteria. Among these, the most common were off-topic papers, review papers, letters to the editor, papers in which the diagnosis of OSA was based on tests other than PSG or the diagnosis of COPD was not consistent with the GOLD criteria, or papers with a small study group (i.e., fewer than 50 subjects). Further verification based on reading the full texts allowed us to exclude another 14 articles because they did not meet the OSA diagnostic criterion, 7 articles because they did not meet the COPD diagnostic criterion, 5 because the full content of the articles was not available in English (i.e., articles in Chinese), and 2 for being off-topic. The result was 38 articles meeting the inclusion criteria [20,21,22,23,24,25,26,27,28,29,30,31,32,33,34,35,36,37,38,39,40,41,42,43,44,45,46,47,48,49,50,51,52,53,54,55,56,57].

### 3.2. Study Characteristics and Study Quality

**Figure 2:**
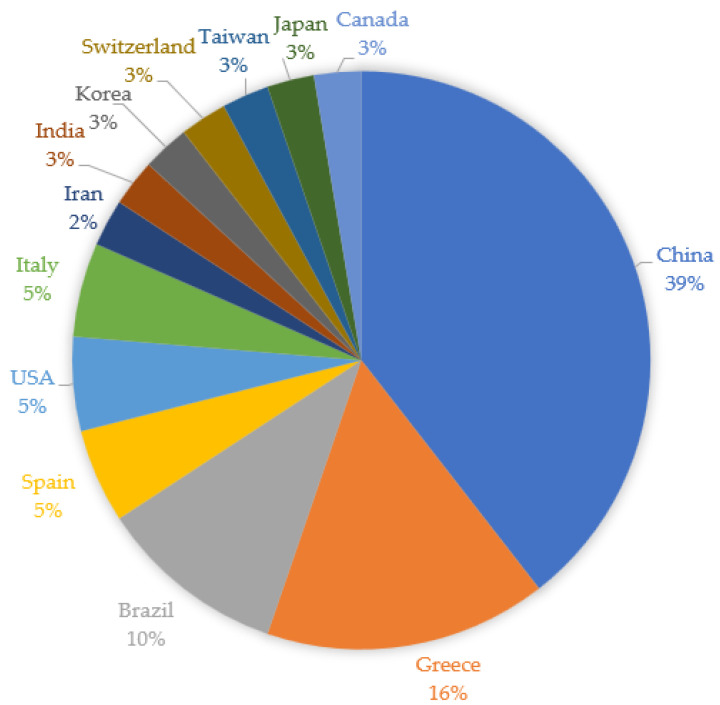
Countries where the selected studies were conducted.

All articles included in this review are original articles published in English concerning the comorbidity of COPD and OSA. The included studies were published in 2022 (4 papers), 2021 (10 papers), 2020 (9 papers), 2019 (9 papers), and 2018 (6 papers). Figure 2 shows which countries the selected studies originated from.

In the included studies, there were a total of 27,064 participants, among whom there were 6515 patients with OS (the patient groups in Cliamaco’s studies [22,23] and Economou’s [56] and Papachatzakis’s [55] studies overlapped, so the numbers of their participants were summed once). The number of studies with specific group size ranges covered by these studies is shown in Figure S1, whereas Figure S2 shows the number of OS patients in the included studies. In the total population of all included studies, 68.1% were men and 31.9% were women. Figure S3 shows the number of papers with the percentage of men in the study populations.

The risk-of-bias scores for each study are shown in Table S2, including the study quality ratings from the Newcastle–Ottawa Scale for cohort and case–control studies [18] and its modified version for cross-sectional studies [19]. In a nutshell, 26 of the included studies were classified as very good, and the remaining 12 as good.

### 3.3. Systematic Review: Broad Overview and Study Characteristics of All Included Studies

Table S3 shows the main information retrieved from all included studies.

#### 3.3.1. Prevalence of Overlap Syndrome

**Figure 3:**
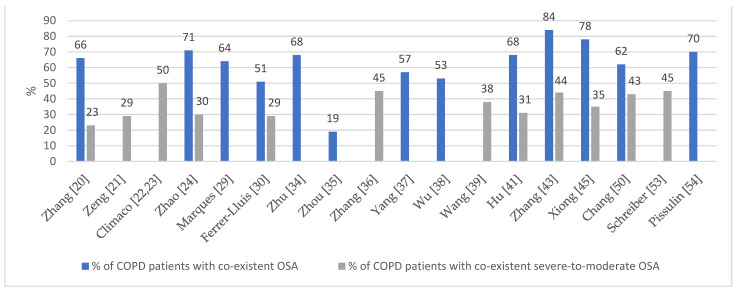
The prevalence of OSA in COPD patients in the included studies [20,21,22,23,24,29,30,34,35,36,37,38,39,41,43,45,50,53,54].

The global prevalence of COPD in the population aged 30–79 years was assessed on average at 10.3% and was the highest in the Western Pacific region (11.7%) and the lowest in the Americas (6.8%) [58]. The prevalence of OSA varies between 9 and 38% [59]. In the included studies, PSG revealed the coexistence of OSA—described as an apnea–hypopnea index (AHI) ≥ 5—in 56.45% to 78% of COPD patients [20,24,29,34,37,41,45,54], which is consistent with the findings of other studies [60,61], but there were also studies where the prevalence of OSA in COPD patients was extremely different, with as few as 19.2% [35] or as many as 84% [43] of COPD patients having coexistent OSA. These discrepancies were due to the different patient populations included in the studies. Moderate or severe sleep apnea (described as AHI ≥ 15) was generally diagnosed in 30–50% of COPD patients [22,24,36,37,39,41,43,45,53]. Figure 3 shows the frequency of OSA comorbidity in COPD patients in the included studies.

Among OSA patients with available spirometry results, the coexistence of COPD was confirmed in 11.9–23.2% [33,44,47,48,62].

There are not enough data to conclude that patients with COPD have a higher risk of developing OSA—both diseases are very common; hence, the incidence of OS may be high [1]. However, the above data indicate the need for awareness of the frequent co-occurrence of OSA in populations of COPD patients.

#### 3.3.2. Meaning of Age and Gender in Overlap Syndrome

Aging and male sex are associated with both COPD [31,34,58,63] and OSA [34,59].

In most of the included studies, no significant differences were observed between the COPD-only and OS patients in terms of age [20,34,36,39,41] and sex [20,34,36,37,39,41,43,45,46,53].

In contrast, most studies found that the OS patients were older than the OSA-only patients [33,47,48,51,57,62] but—similarly to COPD—the gender distribution in both groups was predominately similar, with significant male dominance [22,38,54].

Summarizing these observations, in the OS, symptoms of OSA develop first, followed by COPD, and in all of these diseases the proportion of men is similar.

#### 3.3.3. Relevance of Body Mass Index, Smoking, and Alcohol Consumption in Overlap Syndrome

A body mass index (BMI) less than 18.5 kg/m^2^ is considered to be a risk factor for COPD [58], while a higher BMI increases the prevalence of OSA [59]. It is worth mentioning the existence of the so-called “obesity paradox” phenomenon, which means that among patients with chronic diseases—such as COPD—patients who are overweight or obese have a better prognosis [64,65,66]. While COPD patients with a high BMI have an improvement in their pulmonary function [66], they simultaneously have a higher risk of OSA coincidence and, consequently, the development of OS [20,24,26,34,38,39,41,43,45,53,61,67]. Furthermore, it was estimated that for each additional 1 kg/m^2^ in BMI, the risk of occurrence of OSA in COPD increases 2.552-fold [35].

In conclusion, PSG should be considered particularly in overweight or obese COPD patients, even if they do not present symptoms of sleep apnea, due to their higher risk of developing OS [68].

Current smoking or history of smoking are also known risk factors for COPD [31,58] and OS [69]. There are divergent pieces of information about isolated OSA’s correlation with smoking frequency [70,71,72,73,74] and about its impact on the development of COPD in these patients [20,27,33,36,37,39,41,42,43,45,47,48,57,62].

Regarding the issue of alcohol and the use of antipsychotic drugs, both factors have an impact on sleep architecture in terms of the contribution of rapid eye movement (REM) sleep in COPD patients [29]. In some studies, the coexistence of COPD in OSA patients was correlated with a significantly higher rate of alcohol consumption [48,62].

#### 3.3.4. Sleep Quality and Other Aspects of Quality of Life in Overlap Syndrome

It is important to remember that one of the most common symptoms of OSA is excessive daytime sleepiness. The majority of COPD patients report poor sleep quality, which is a significant predictor of poor QoL [75,76,77,78]. COPD patients with poor sleep quality are suggested to have higher anxiety and depression scores than COPD patients who are classified as good sleepers [79]. Additionally, the severity and exacerbation of COPD might be associated with poor sleep quality [80], as a result of symptoms such as wheezing, phlegm, or inhaled corticosteroid use [81]. In OS patients, poor sleep quality is similar [22] or even worse [41] than in COPD-only patients.

The majority of the included studies found higher results on the Epworth sleepiness scale (ESS) in OS compared to patients with COPD alone [20,34,39,41]; however, taking into consideration the fact that in other studies only about 30–40% of patients with an OS diagnosis had confirmed OSA based on ESS scores [53,82], it seems that the ESS is not a reliable questionnaire for identifying OS patients in COPD patient populations.

The researchers also assessed other aspects of QoL in OSA, including persistent morning headaches, morning tiredness, daytime sleepiness, exertional dyspnea, and fatigue (fatigue severity scale—FSS) [56], but also nocturia [62] and erectile dysfunction [33], which were more common when OSA was accompanied by COPD [33].

#### 3.3.5. Polysomnography Findings and Pulmonary Function in Overlap Syndrome

In the included studies, there was a lot of discrepancy in the PSG results when OS patients were compared to COPD-only patients. As expected, most studies showed that OS patients had a lower percentage of total sleep time (TST) and sleep time in the REM phase and non-rapid eye movement (NREM) stage 3, but higher sleep time in NREM stage 1 and a higher arousal index compared to the COPD-only group. Moreover, most commonly, in PSG examinations, investigators observed higher AHI, hypopnea index, and oxygen desaturation index (ODI), but lower nadir and mean oxygen saturation (SaO_2_), when COPD was associated with OSA [21,22,23,24,34,35,36,37,39,41,43,45,46,51,54].

Similar to the situation above, there was a lot of discrepancy in the PSG results when OS patients were compared to OSA-only patients. However, most researchers agree that the AHI in OS patients is comparable to the OSA-only group, but additional COPD diagnosis in OSA patients causes additional oxygen desaturation during sleep [51,55,57,62,83]. Additionally, AHI, TS90% (TST with SaO_2_ below 90%), vital capacity (VC), and FEV_1_/FVC were the strongest predictors of hypercapnia among OS patients [49,52]. Moreover, TS90% increased with the severity of both OSA and COPD [30].

In most of the studies, no significant differences were found in pulmonary function parameters between OS and COPD-only patients [36,39,43], nor in the GOLD classification [26]. In contrast, as expected, the comparison of the OS group to OSA-only patients showed significantly lower values of FEV_1_/FVC, FEV_1_, FVC%, peak expiratory flow, and SaO_2_ in the first group [55,56,62]. However, the severity of COPD did not have an impact on the severity of OSA in patients with an OS diagnosis [27,38,84]. On the other hand, unrecognized and untreated OSA increased hospital readmissions in patients admitted for COPD exacerbation [24,34,85,86,87], but treatment with continuous positive airway pressure (CPAP) in OS patients decreased the risk of COPD exacerbation to frequencies similar to those in COPD-only groups [14].

#### 3.3.6. Blood Test Results in Overlap Syndrome

A. Complete blood counts

Many researchers confirm that OS may influence the blood results of patients.

In COPD patients, platelet–lymphocyte ratio and neutrophil–lymphocyte ratio were found to be significantly elevated during exacerbation and were positively correlated with smoking index, COPD stage, and dyspnea severity [88]. Blood eosinophilia was suggested to be a predictor for increased risk of future exacerbations in COPD patients [89].

Moreover, COPD, OSA, and OS patients all showed an increase in markers of platelet activation (e.g., PDW (platelet distribution width) or MPV (mean platelet volume)), which is associated with a higher risk of cardiovascular (CV) events [57,90,91,92].

The components of OS, OSA, and COPD can lead to chronic hypoxia, causing red blood cell (RBC) proliferation and, potentially, polycythemia [34,93,94,95]. Therefore, continuous or nocturnal supplemental oxygen use is associated with a decreased risk of polycythemia among COPD patients [95,96]. Most research confirms that polycythemia is more common in patients with OS than those with COPD only, and that hemoglobin and RBC counts increase with the severity of OSA in COPD patients [21,34].

B. Metabolic and biochemical results

Some studies assessed the metabolic and biochemical results in OS patients. They found that OS patients had higher levels of fasting glucose, fasting insulin, glycated hemoglobin (HbA1c), total cholesterol, low-density lipoprotein cholesterol (LDL-C), triglycerides (TG), leptin, resistin, and adiponectin compared to COPD-only patients, whereas high-density lipoprotein cholesterol (HDL-C) was significantly lower in the OS groups [26,35]. Compared to COPD-only or OSA-only patients, OS patients had significantly lower partial pressure of oxygen (PaO_2_) and arterial SaO_2_, but higher partial pressure of carbon dioxide (PaCO_2_) and serum levels of B-type natriuretic peptide. Moreover, OS patients had higher serum levels of D-dimer, cardiac troponin T, and lactate dehydrogenase than OSA-only patients (*p* < 0.001) [26].

C. Inflammatory indicators

Taking into account inflammatory indicators, the studies demonstrated that OS patients had significantly higher levels of C-reactive protein (CRP), interleukin-6, percentage of peripheral neutrophils, levels of soluble vascular cell adhesion molecule-1 (sVCAM-1), and tumor necrosis factor α (TNFα) compared to COPD-only patients [26,35,46,97,98]. Higher levels of inflammatory biomarkers were associated with lower physical activity in OS patients [97]. In contrast, the percentages of CD4+ and CD4+/CD8+ lymphocytes were significantly lower in the OS group than in the healthy subjects, OSA-only, and COPD-only groups. These data indicate more severe vascular injury, stronger inflammatory response, and lower cellular immune function in OS patients [46].

Another inflammatory factor assessed in OS patients was soluble receptor for advanced glycation end products, whose levels were reduced in OSA and COPD patients but improved after treatment with CPAP [99]. Recently, attention has been drawn to the role of extracellular vesicles as diagnostic and therapeutic biomarkers in COPD [100] and OSA [101], but their significance in OS has been not investigated yet.

In summary, much attention is paid to searching for OS-specific biomarkers. The high probability of concurrent elevations of HbA1c, CRP, and EPO levels should indicate a high suspicion of OSA and might be correlated with OS and its severity [102,103].

#### 3.3.7. The Role of Comorbidities in Overlap Syndrome

Results from the included studies indicate that the burden of comorbidities in OS is greater than in those with only OSA or COPD. Co-occurrence of multiple diseases (at least four) was more frequently observed in OS than in OSA-only patients (29% vs. 10.5%), and it was especially evident for cardiovascular disease (CVD) [25,55]. Apart from CVD, the most prevalent comorbidities in OS and OSA-only patients were hypertension, diabetes mellitus (DM), dyslipidemia, and depression [25,55]. Moreover, the OS patients had higher Charlson comorbidity index (CCI) scores and probability to suffer concomitant pulmonary thromboembolism, hypercapnia, and/or respiratory failure than COPD- or OSA-only patients [26,104]. Furthermore, OS was independently associated with the prevalence of PE, which may be related to its high hypoxic burden [105]. However, the predisposition to other vascular diseases in COPD, OSA, and OS is unclear. We have divergent data on the prevalence of stroke and arteriopathy [26,33,41,48,62].

#### Hypertension, Coronary Heart Disease, and Cardiovascular Diseases in Overlap Syndrome

**Figure 4:**
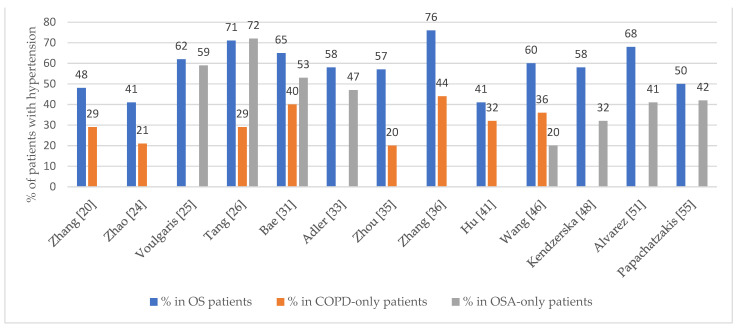
The prevalence of hypertension in OS, COPD-only, and OSA-only patients in the included studies [20,24,25,26,31,33,35,36,41,46,48,51,55].

In most studies, the OS diagnosis significantly increased the risk of developing hypertension compared to COPD alone [20,24,26,31,35,36,41,46,48,83] or OSA alone [31,33,46,48,51,106] (Figure 4). On the other hand, OSA was found to be an independent risk factor for hypertension [41]. Figure S4 (Supplementary Materials) presents the occurrence of hypertension in OS patients in the included studies.

Coronary heart disease (CHD), including myocardial infarction, was significantly more prevalent in OS patients than in COPD-only and OSA-only groups [20,24,26,31,33,39,46,48] (Figure S5).

As mentioned above, COPD and OSA were both associated with an increased risk of CVD, and the combination of these two diseases in OS increased the risk even more [41,47,83,107] (Figure S6).

The most important pathophysiological triggers for CVD observed in OS were intermittent hypoxia, recurrent arousals, and intrathoracic pressure swings [108]. The OS patients showed significantly higher irregularity of overnight pulse and greater common carotid artery stiffness than COPD-only or OSA-only patients, which could lead to cor pulmonale [20,23,26,51,109,110,111,112].

According to the literature, there is also a high prevalence of COPD and OSA among patients with atrial fibrillation (AF) [113,114,115]. Some studies [26,116] showed that OS patients had more AF incidents than COPD-only or OSA-only patients, as confirmed by PSG, where AF events were observed in 24% of OS patients [32].

From the practical point of view, early diagnosis of OS and appropriate treatment may reduce CV risk and prolong survival in this group of patients [117]. It was suggested that untreated OS might cause more extensive right-ventricular remodeling than COPD alone, and that the extent of right ventricular remodeling was associated with the severity of oxygen desaturation [118]. Some studies reported that CPAP treatment reduced pulse wave velocity in OSA patients [119]. However, unfortunately, in randomized trials, CPAP treatment has not shown the ability to improve CV outcomes [120].

#### Role of Diabetes and Metabolic Syndrome in Overlap Syndrome

In most of the included studies, OS patients more frequently suffered from DM than COPD-only patients [20,26,39], but there were varied opinions as to whether DM had a higher prevalence in the OS groups than in OSA-only patients [25,48,55,83] (Figure S7).

It has been noted that COPD patients with coexistent DM are at higher risk of severe exacerbations and death, because DM affects the progression of this disease as well as the CV risk [121]. For this reason, appropriate antidiabetic treatment is especially important in this group [122].

OS patients were characterized by worse metabolic results—including higher detection rates of metabolic syndrome (MS), abdominal obesity, high blood pressure, high TG, low HDL-C, and high glucose levels—compared to COPD-only and OSA-only patients [35,42,123,124]. Unsurprisingly, BMI and age emerged as independent predictors of MS in OS patients [42]. Collectively, these results imply that early identification and treatment of MS may play a significant role in the prevention of complications related to OS [42].

##### Depression, Anxiety, and Cognitive Function in Overlap Syndrome

It is known that depression is more common in COPD patients compared with general society [125], and the coexistence of anxiety or depression among COPD patients is associated with a higher risk of COPD exacerbations [126,127,128]. Additionally, the same increased risk of depressive disorders was also confirmed among OSA patients and was particularly evident in women [129]. The occurrence of depression was significantly related to the severity of OSA, suggesting that it is an independent risk factor for depression [24]. Thus, a lot of studies focused on the assessment of depression and anxiety in OS. The included studies demonstrated that, compared to pure COPD patients, more patients in the OS group had depression (54% vs. 38%) and anxiety (77% vs. 23%) [20,24]. Unlike the above observations, the degree of anxiety and depression was similar in OS and OSA-only patients [56]. Luckily, CPAP treatment seems to be effective in reducing the prevalence of depression and improving QoL in OS and OSA patients [33,130].

Depression and anxiety observed in OS patients could influence their cognitive function. Both OSA and COPD are associated with cognitive impairment in attention, memory, executive function, psychomotor function, and language abilities, linked with hypoxia and hypercarbia [39,131]. In OS patients, cognitive dysfunction might be also associated with more frequent smoking [132], changes in somatotropic axis hormones [133], and a history of COPD exacerbations [134].

The OS patients had lower executive function processing speed compared to COPD-only or OSA-only patients. This difference was suggested to be associated with more intense vascular problems and elevated common carotid artery stiffness in the OS group, leading to cerebral ischemia [109]. The assessments in the mini mental state examination (MMSE) and Montreal cognitive assessment score, which are used for the evaluation of cognitive function [135], were significantly worse in OS than in COPD-only patients [36,39]. About 66% of OS patients had a risk of dementia, compared to 31% of COPD-only patients [36]. Risk factors for dementia included older age, lower educational level, and higher AHI and ODI values, and three of them—age, educational level, and ODI—were independent variables [36]. Moreover, the level of dementia in OS was also significantly correlated with the severity of OSA [36].

#### 3.3.8. Risk of Mortality in Overlap Syndrome

COPD, OSA, and OS patients are at greater risk of all-cause mortality compared with the general population. Even short respiratory events are connected with increasing ventilatory instability and augmented autonomic nervous system responses [136].

There is divergent information as to whether the coexistence of OSA and COPD is associated with a higher likelihood of death than each of them alone. Most studies confirmed that OS was associated with a higher all-cause mortality rate than OSA alone, but the mortality risk was comparable for OS and COPD-only patients [31,137,138]. The 1-year follow-up recorded 21.5% mortality in OS patients, 7% in COPD-only patients, and 10.1% in OSA-only patients [26]. Moreover, the CCI score, hypertension, pulmonary thromboembolism, and heart failure were identified as independent predictors of all-cause mortality [26]. Another independent risk factor of mortality in OS patients is AF [32].

Subsequent studies have focused on evaluating the effects of CPAP treatment on mortality outcomes. They showed that OS patients treated with CPAP had improved survival with no increased risk of death compared to COPD-only patients [14]. In another study on a large cohort of military veterans with an OSA diagnosis, who were not treated with CPAP, the average risk of death was 1.34 times higher than for those undergoing treatment. In OSA patients who were non-adherent to CPAP therapy, the average adjusted risk of death was as much as 1.78 times higher compared to those using it at least 70% of nights and for more than 4 h nightly [139].

In summary, in OS patients, a higher rate of mortality was significantly associated with respiratory problems such as AFL, higher AHI, and lower time of CPAP use [49], but also with CV problems such as hypertension, pulmonary thromboembolism, heart failure, and AF. Therefore, it is very important to monitor CV and lung functions and to ansure good compliance with CPAP treatment.

#### 3.3.9. Treatment in Overlap Syndrome

In selected publications, some researchers assessed the impacts of the applied pharmacological treatment and CPAP therapy on the course of the disease. The studies showed that OS patients treated with CPAP had a lower risk of death and hospitalization because of COPD exacerbations [14,140]. After 1 year of CPAP treatment in OS patients, significant improvements were observed in PaO_2_ (median value: 65 vs. 71 mmHg) and PaCO_2_ (39.8 vs. 38.3 mmHg)—especially in the subgroup of hypercapnic patients [52]. In OS patients with an FEV_1_ lower than 79.1%, there was a significant improvement in FEV_1_, but in OS patients with an FEV_1_ higher than 79.1%, there was a significant worsening of FEV_1_ [52]. In a real-world data study, CPAP treatment in OS patients led to reductions in all-cause emergency room visits and hospitalizations, severe acute exacerbations, and healthcare costs [141].

Other authors confirmed that CPAP treatment improved the overall burden of symptoms related to sleep apnea in OS and OSA-only patients [33]. However, 3 months of CPAP treatment performed in OS patients was not enough to impact the degree of sleepiness (ESS), depression and anxiety (HADS—hospital anxiety and depression scale), or fatigue (FSS) [56].

Based on the reported research, the availability of CPAP therapy varies between patients. Patients with OS were more likely to receive CPAP therapy than those in the OSA-only group [26]. Another aspect of CPAP treatment is a problem with the patient’s adaptation to this therapeutic method, which affects the final results [33,49]. Furthermore, in OS patients, non-adherence to CPAP treatment was explained by claustrophobia (17%), poor mask fit (38%), and excessive air leakage (11%) [49].

Some authors assessed differences in the pharmacological treatments applied for OS patients and COPD-only patients. A few of them did not notice discrepancies [41], but others [26] mentioned that antisterone, nifedipine, diuretics, angiotensin-converting enzyme inhibitors, angiotensin receptor blockers, beta blockers, and aspirin were more commonly used in OS patients [26].

##### Tools for Identifying Overlap Syndrome Patients

The authors compared the performance of various tools in screening for OSA in populations of COPD patients. As a result, they concluded that ODI had the best accuracy in identifying OSA in chronic respiratory diseases, compared with the STOP-BANG questionnaire (SBQ), Berlin questionnaire (BQ), and ESS [43]. Furthermore, the SBQ—especially with a cutoff threshold of ≥6—has higher sensitivity for detecting OSA than the BQ, STOP questionnaire, and ESS; thus, the SBQ was indicated as a tool that should be used by clinicians to screen for patients at risk of OSA in COPD populations [38,43,142,143,144]. The BQ showed good sensitivity (0.83) for the recognition of OSA in COPD patients, but low specificity (0.32) and accuracy (0.63), so it is not a good enough tool to identify OS patients [54]. To predict the coexistence of severe OSA in COPD patients, some authors proposed a simplified screening questionnaire [40,145]. One of them was based on snoring (1 point), witnessed apnea (2 points), BMI ≥ 27.5 kg/m^2^ (1 point), and CHD (1 point). Using a cutoff threshold of ≥2/5 for AHI ≥ 30, the questionnaire had a sensitivity of 85.2% and specificity of 80.4% in the validation group [40].

Other authors demonstrated the utility of the Nox-T3 portable monitor for the diagnosis of OSA in COPD patients by comparing it to PSG, confirming that this device can be used to examine this group of patients [50]. Additionally, another portable diagnostic device—WatchPAT (Itamar Medical, Franklin, Massachusetts, USA)—was able to determine OSA in COPD patients with good sensitivity [146].

## 4. Discussion

The term OS, or COLDOSA, was first coined by David C. Flenley in 1985 to describe the coexistence of OSA in patients with COPD [147]. Combined, these conditions cause a significant drop in oxygen during sleep, leading to an increased risk of disability and death. They are two common and separate conditions, and both of them affect the airways in different ways.

COPD is usually caused by smoking and is characterized by chronic inflammation that leads to progressive obstruction of airflow to the alveoli in the lungs, whereas OSA is caused by intermittent upper-airway collapse during sleep. Keeping in mind that patients with OS have an increased risk of mortality due to CV events, it is crucial for clinicians to evaluate patients with OSA or COPD for the occurrence of OS and provide effective treatment.

OS should be seen as a common syndrome to be treated. Due to the difficulty of performing PSG on patients with COPD, the diagnosis of comorbid OSA is often missed and patients do not receive appropriate treatment, increasing their risk of suffering from CV and cerebrovascular diseases [30]. A major factor that we need to consider when approaching OS is that both COPD and OSA are significantly underdiagnosed, so OS diagnosis is underestimated. The true prevalence of both COPD and OSA in adults over 40 is thought to be between 5 and 10%. From a clinical point of view, screening all patients with severe COPD for OSA might be appropriate, because CPAP therapy or surgical treatment for OSA [148] should be offered to patients with OS due to the expected significant improvement in OSA-related symptoms, although the range of response may be less dramatic than in OSA [33].

The included studies are of great value in understanding the relationships associated with the co-occurrence of COPD and OSA, paving the way for further research in this area. Their main strengths are that many of the studies were conducted on large samples [20,21,24,25,32,34,41,42,45,48,49,52], and all diagnoses of OSA were established by overnight PSG. It was shown that the diagnosis of OSA cannot be based only on nocturnal home oximetry, even if typical cyclical changes in saturation are observed by oximetry tracing [149]. Moreover, overnight PSG is still the gold standard for OSA diagnosis and should be performed if there is a suspicion of OSA in COPD patients. The included studies used strict protocols and provided detailed characterizations of individuals.

However, we also found a few limitations in the presented research. Firstly, the vast majority of the included studies were retrospective [26,30,31,32,40,49] and cross-sectional studies [20,21,22,23,24,25,27,28,29,34,35,36,37,38,39,41,42,43,44,45,46,47,50,51,55], with the study design implying that it was not possible to look for causal relationships between the correlations found. Secondly, some analyses included a small study sample and were single-center studies [22,23,27,36,46,50,52,54,55,56]. Furthermore, the diagnosis of OSA was established with different thresholds of AHI—sometimes AHI ≥ 5 [24,25,27,28,30,32,34,35,37,38,41,42,44,45,46,47,49,50,54,55,56,57], and in others AHI ≥ 15 [20,21,22,23,31,33,36,39,43,53] or even AHI ≥ 10 [52]—and this difference made comparing particular studies more difficult. According to guidelines [15], the diagnosis of OSA is made based on the occurrence of symptoms and AHI ≥ 5 in PSG, or AHI ≥ 15 in the absence of sleep-related symptoms. The authors who chose the limit AHI ≥ 15 probably aimed to analyze only patients with moderate-to-severe OSA. Moreover, we only reviewed papers from January 2018 onward, because we focused on the most recent reports so that we could skip other relevant but older studies. Furthermore, we only included studies in which the diagnosis of OSA was made based on PSG, meaning that many very interesting studies based on polygraphy were not included in this systematic review. Additionally, it should be noted that the study groups were not always representative of the population of each disease entity. Some of the studies were conducted on patients hospitalized for COPD [34,36,41,43,45], undergoing inpatient pulmonary rehabilitation programs [53], referred to PSG studies for sleep disorders [24,27,28,31,33,40,42,46,47,48,49,50,51,57], referred to spirometry because of symptoms [31,33,44,46], or who applied for an open invitation for participation in the study [36,38,39,54]. Furthermore, in a few studies, OS patients were not representative of the total OS population but were extremely commonly selected, which also might have had an impact on the studies’ outcomes [27,33]. Moreover, the problem of the availability of PSG data and difficulty in performing sleep tests among patients with severe COPD (GOLD 4) was pointed out, because this group of patients is usually chronically treated with oxygen therapy [30,33]. Furthermore, in a lot of studies, not all COPD patients underwent PSG—only patients with higher suspicion of OSA based on ESS scores; thus, these studies did not describe the accurate frequency of OS diagnosis in the group [28]. It should also be mentioned that the value of ESS in the prediction of OSA in COPD patients was assessed as poor [45]. Finally, due to the heterogeneous inclusion criteria, the results should be compared with caution. Importantly, most of the studies did not have control groups including healthy patients [20,21,22,23,24,25,26,27,28,29,33,34,35,36,37,38,40,41,43,48,49,51], OSA-only patients [20,21,22,23,24,27,28,29,34,35,36,37,38,41,43,49], and COPD-only patients [25,27,33,47,48,49], further reducing the reliability of the obtained results. Another limitation is that in the majority of the studies PSG was conducted during only a single night, preventing us from excluding the significance of internight variability in AHI [150]. Significant differences were noted in respiratory events at the intrapersonal level in subjects who underwent more than one sleep study. These differences may influence the misdiagnosis or classification of patients based on only a single diagnostic sleep study [151].

Based on the scientific data presented, it seems necessary to conduct appropriate screening for OSA among patients with COPD. The included studies can be regarded as preliminary studies in which some correlations were observed, but further research is needed to understand the cause of these correlations—especially if they are extremely different from one another. There is still a need for further research concerning OS, because many questions remain unanswered [1]. Moreover, it is essential to conduct other studies that could demonstrate whether treatment with CPAP influences the prevalence of comorbidities in OS patients, as well as whether this therapy could reduce the cardiometabolic risk [25], effect on MS components, and systemic inflammatory profile in OS [35].

## Figures and Tables

**Table 1:** Inclusion and exclusion criteria for the retrieved studies.

Inclusion Criteria	Exclusion Criteria
Completed, published	Unfinished, unpublished
Original articles	Reviews, letters to the editor, conference papers, case reports, book chapters, expert opinions
Observational studies	Experimental studies
Full text available in English	Language other than English or only abstract available in English
Human studies	Animal studies
Studies concerning the OS	Studies not related to the OS
Diagnosis of COPD by GOLD guidelines [4]	Diagnosis of COPD not matching the GOLD criteria [4]
Diagnosis of OSA based on PSG	Diagnosis of OSA based on other sleep studies than PSG, such as polygraphy or a questionnaire survey
Good-quality studies	Poor-quality studies
Studies published from January 2018 to 26 October 2022	Studies published before January 2018
At least 50 participants	Fewer than 50 participants

## Data Availability

Not applicable.

## Supplementary Materials

The following supporting information can be downloaded at: https://www.mdpi.com/article/10.3390/biomedicines11010016/s1, Table S1: Databases and the search strategy; Table S2: Risk of bias assessed using the Newcastle–Ottawa quality assessment scale for cohort and case–control studies, along with its modified version adapted for cross-sectional studies; Table S3: Main information obtained from the retrieved studies; Figure S1: The size of the study population in the included studies; Figure S2: The number of OS patients in the included studies; Figure S3: Percentage of men in the study populations in the included studies; Figure S4: The prevalence of hypertension in OS patients in the included studies; Figure S5: The prevalence of CHD in OS patients in the included studies; Figure S6: The prevalence of CVD in OS patients in the included studies; Figure S7: The prevalence of DM in OS patients in the included studies. References [20,21,22,23,24,25,26,27,28,29,30,31,32,33,34,35,36,37,38,39,40,41,42,43,44,45,46,47,48,49,50,51,52,53,54,55,56,57] are cited in the supplementary materials.

## Abbreviations

AF	Atrial fibrillation
AFL	Airflow limitation
AHI	Apnea–hypopnea index
BMI	Body mass index
BQ	Berlin questionnaire
CCI	Charlson comorbidity index
CHD	Coronary heart disease
COPD	Chronic obstructive pulmonary disease
CPAP	Continuous positive airway pressure
CRP	C-reactive protein
CV	Cardiovascular
CVD	Cardiovascular disease
DM	Diabetes mellitus
ESS	Epworth sleepiness scale
FEV_1_	Forced expiratory volume in 1 s
FVC	Forced vital capacity
FSS	Fatigue severity scale
GOLD	Global Initiative for Obstructive Lung Disease
HADS	Hospital anxiety and depression scale
HDL-C	High-density lipoprotein cholesterol
HbA1c	Glycated hemoglobin
MS	Metabolic syndrome
NREM	Non-rapid eye movement
ODI	Oxygen desaturation index
OS	Overlap syndrome of chronic obstructive pulmonary disease and obstructive sleep apnea
OSA	Obstructive sleep apnea
PaO_2_	Partial pressure of oxygen
PaCO_2_	Partial pressure of carbon dioxide
PRISMA	Preferred Reporting Items for Systematic Reviews and Meta-Analyses
PSG	Polysomnography
RBC	Red blood cell
REM	Rapid eye movement
SaO_2_	Oxygen saturation
SBQ	STOP-BANG questionnaire
TG	Triglycerides
TS90%	Percentage of total sleep time with SaO_2_ below 90%
TST	Total sleep time
VC	Vital capacity
QoL	Quality of life
