# Supplementary material for: The Overlap Syndrome of Obstructive Sleep Apnea and Chronic Obstructive Pulmonary Disease: A Systematic Review
Source: Biomedicines. 2022 Dec 21;11(1):16. doi: 10.3390/biomedicines11010016 (PMC9856172; doi:10.3390/biomedicines11010016)
Supplement: Supplementary file 1 [file biomedicines-11-00016-s001.zip › biomedicines-2065695-supplementary.pdf]

Table S1. Databases and the search strategy.

Database	Search Strategy	Number of Results
PubMed/MEDLINE	"Sleep Apnea, Obstructive"[Mesh] AND "Pulmonary Disease, Chronic Obstructive"[Mesh]<br>Limited to: Publication Years 2018-2022	146
Scopus	TITLE-ABS-KEY ((chronic obstructive pulmonary disease) OR (chronic obstructive lung disease) OR (chronic obstructive airway disease) OR (COAD) OR (COPD)) AND ((obstructive sleep apnea) OR (OSA) OR (sleep apnea syndrome))<br>Limited to<br>1. Document type: Article<br>2. Language: English<br>3. Publication Years 2018-2022	1373
Cochrane	#1 MeSH descriptor: [Sleep Apnea, Obstructive] explode all trees<br>#2 MeSH descriptor: [Pulmonary Disease, Chronic Obstructive] explode all trees<br>#1 AND #2	29
3 databases		1548

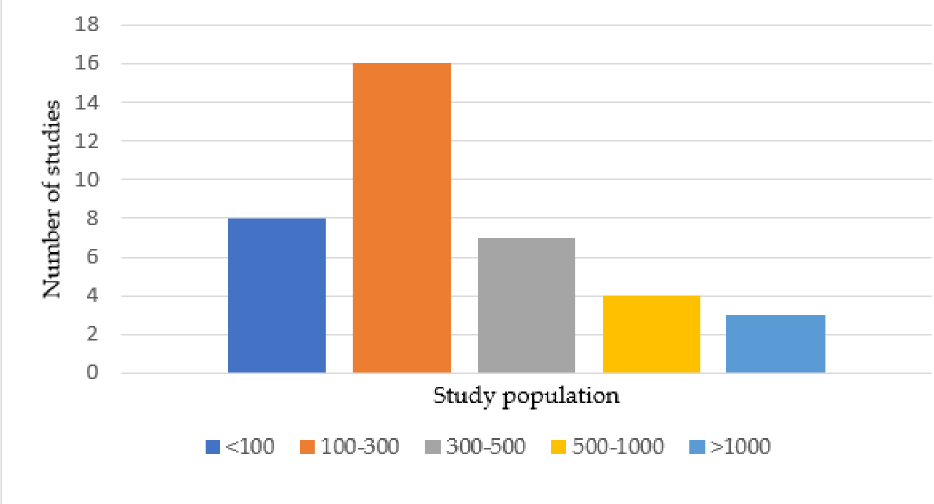

Figure S1. The size of the study population in the included studies.

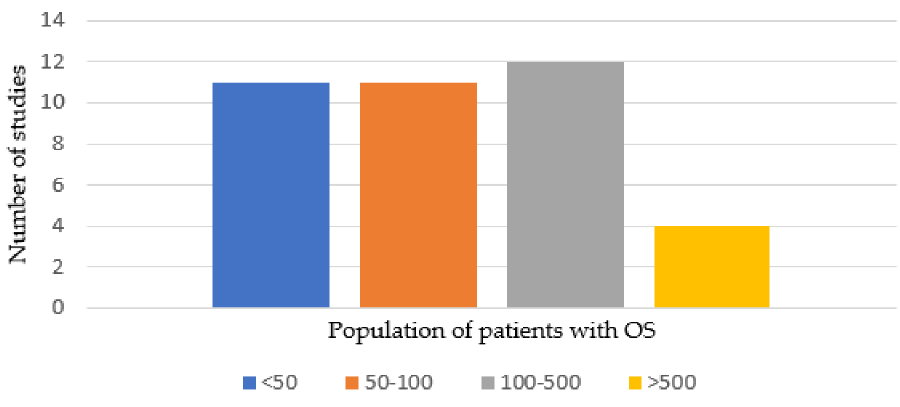

Figure S2. The number of OS patients in the included studies.

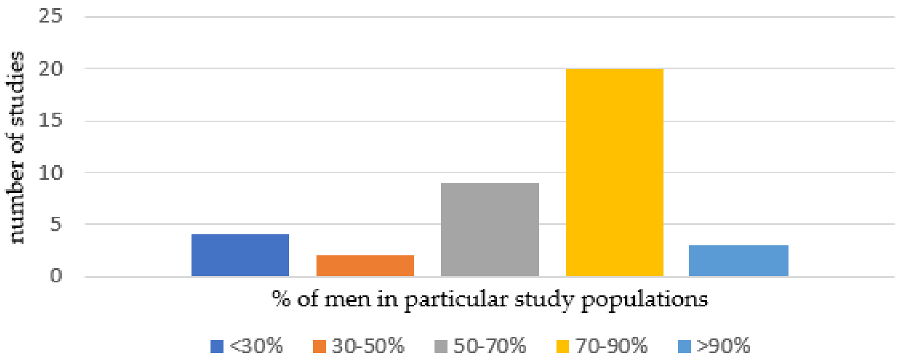

Figure S3. Percentage of men in the study population in the included studies.

Table S2. Risk of bias assessed using the Newcastle-Ottawa quality assessment scale for cohort and case-control studies and its modified version adapted for cross-sectional studies. A total score of 0-3 was considered unsatisfactory, 4-5 points satisfactory, 6-7 points good, and 8-9 points very good.

**Study**	**Selection** (max score of 4)	**Comparability** (max score of 2)	**Outcome** (max score of 3)	**Total score** (max score of 9)
Zhang 2022 [20]	4	2	2	8
Zeng 2022 [21]	4	1	2	7
Climaco 2022 (a) [22]	4	1	2	7
Climaco 2022 (b) [23]	4	1	2	7
Zhao 2021 [24]	4	2	2	8
Voulgaris 2021 [25]	4	2	2	8
Tang 2021 [26]	4	2	2	8
Sami 2021 [27]	3	2	2	7
Nattusami 2021 [28]	3	2	3	8
Marques 2021 [29]	4	2	1	7
Ferrer-Lluis 2021 [30]	4	2	2	8
Bae 2021 [31]	4	2	2	8
Akinnusi 2021 [32]	4	1	2	7
Adler 2021 [33]	3	2	3	8
Zhu 2020 [34]	4	2	2	8
Zhou 2020 [35]	3	2	3	8
Zhang 2020 [36]	4	2	3	9
Yang 2020 [37]	4	2	2	8

Wu 2020 [38]	4	2	3	9
Wang 2020 (a) [39]	4	2	2	8
Wang 2020 (b) [40]	4	2	2	8
Hu 2020 [41]	4	2	3	9
Archontogeorgis 2020 [42]	3	2	3	8
Zhang 2019 [43]	3	2	3	8
Yamaguchi 2019 [44]	3	2	3	8
Xiong 2019 [45]	4	2	2	8
Wang 2019 [46]	2	1	3	6
Voulgaris 2019 [47]	3	2	3	8
Kendzerska 2019 [48]	3	2	3	8
Jaoude 2019 [49]	3	2	2	7
Chang 2019 [50]	3	1	3	7
Alvarez 2019 [51]	4	2	3	9
Schreiber 2018 (a) [52]	3	1	3	7
Schreiber 2018 (b) [53]	3	1	3	7
Pissulin 2018 [54]	3	2	3	8
Papachatzakis 2018 [55]	3	2	3	8
Economou 2018 [56]	3	1	3	7
Archontogeorgis 2018 [57]	3	2	3	8

Table S3. Main information obtained from the retrieved studies.

Study	Study Design	Study Population	Main aim	Main results
Zhang 2022 [20]	Cross-sectional study	COPD patients (n=842), among them OS patients (n=194)	To investigate the prevalence and possible predictors of OSA coexistence in COPD patients and its effect on clinical outcomes.	• 66% of investigated COPD patients with AHI ≥ 5.<br>• No significant differences ($p>0.05$) between only COPD and OS groups in age, sex ratio, modified Medical Research Council dyspnea scale score, smoking index, number of acute exacerbations and hospitalizations in the last year, and prevalence of cor pulmonale.<br>• In the OS group relative to only the COPD group significantly higher BMI, neck circumference, CAT score, CCI, ESS, HADS, and SBQ scores, FEV_1, FEV_1 %pred, FEV_1/FVC ratio, the prevalence of hypertension, CHD, and DM.<br>• In the OS group relative to only the COPD group significantly lower ($p<0.05$) prevalence of severe COPD.<br>• Independent risk factors for OSA in COPD patients ($p<0.05$): BMI, neck circumference, ESS, CAT, CCI, HADS, hypertension, and DM.<br>• Patients with severe COPD have a lower risk of OSA than mild or moderate COPD patients.
Zeng 2022 [21]	Cross-sectional study	COPD patients (n=886), among them OS patients (n=264)	To evaluate the prevalence of polycythemia in OS patients and analyze the impact of OSA on polycythemia.	• Polycythemia was significantly more frequent in OS patients (6.4%) than COPD alone patients (2.9%) ($p < 0.05$).<br>• The prevalence of polycythemia increased with OSA severity in all patients with GOLD grade 1-2 COPD, but not in GOLD grade 3-4 COPD patients.<br>• TS90% was independently associated with increased odds of polycythemia and increased Hb (especially in 1-2 GOLD grade patients)
Climaco 2022 [22]	Cross-sectional study	COPD patients (n=102), among them OS patients (n=51)	To evaluate clinical predictors of poor sleep quality in COPD and OS patients	• Poor sleep quality was observed in 74.8% of COPD patients with no significant difference between patients with and without coexisting OSA regarding the Pittsburgh Sleep Quality Index scores ($p=0.73$).<br>• In the group of OS patients, there were observed increased stage 1 NREM sleep and AI, and reduced sleep efficiency and stage 3 NREM sleep ($p<0.05$).<br>• Independent predictors of poor sleep quality were GOLD grade C/D COPD, a CAT score ≥10, and the lowest SaO_2 <80%.
Climaco 2022 [23]	Cross-sectional study	COPD patients (n=102), among them OS patients (n=51)	To evaluate arterial stiffness using PWV in patients with COPD and OS.	• High PWV values were present in 42% of study patients.<br>• No statistical differences between COPD-only and OS patients in PWV values, central BP, peripheral BP, and augmentation index ($p>0.05$).

Zhao 2021 [24]	Cross-sectional study	COPD patients (n=252), among them OS patients (n=180)	To evaluate the prevalence and identify influencing factors of anxiety and depression in OS patients.	• Among OS patients 54% had depression, and 77% had anxiety.<br>• Compared to COPD-only patients, OS patients had higher anxiety ($p=0.018$) and depression ($p=0.022$) scores.<br>• Compared to COPD-only patients, OS patients more frequently had coexistent hypertension (21% vs 41%) and CHD (4% vs. 14%).<br>• Independent risk factors for depression were chest pain, CAT score, and OS ($p<0.05$).<br>• Anxiety and depression positively correlated in the study group ($p<0.001$).
Voulgaris 2021 [25]	Cross-sectional study	OS patients (n=163), OSA-only patients (n=163)	To investigate the prevalence of comorbidities in OSA alone and OS patients.	• No statistical differences in terms of AHI and ODI between OS and OSA-only patients.<br>• OS patients had lower average SaO_2 and higher sleep time with oxygen saturation <90% during sleep, and lower PaO_2 and higher $PaCO_2$ in wakefulness relative to OSA-only patients.<br>• OS patients were characterized by a higher prevalence of total comorbidities than OSA-only patients, which was caused by the higher prevalence of CVD. No differences were observed in other comorbidities.<br>• The most prevalent comorbidities for both OS and OSA-only were arterial hypertension, dyslipidemia, CVD, and DM.
Tang 2021 [26]	Retrospective cohort study	OS patients (n=79), COPD-only patients (n=158), OSA-only patients (n=158)	To evaluate the burden of CVD and long-term outcomes in OS patients.	• OS patients with a higher risk for PH, heart failure, and all-cause mortality than patients with COPD or OSA alone ($p<0.05$).<br>• Hypertension, pulmonary thromboembolism, and heart failure were found to be independent risk factors for 1-year all-cause mortality.
Sami 2021 [27]	Cross-sectional study	OS patients (n=68)	To compare PSG findings of OS patients according to the severity of lower airway obstruction.	• N2 sleep stage was significantly longer in OS patients with FEV_1 <50% than in patients with FEV_1 ≥50% ($p=0.039$).<br>• Other PSG findings (except the N2 stage) were not different in OS patients according to the severity of lower airway obstruction.
Nattusami 2021 [28]	Cross-sectional study	COPD patients (n=301), among them patients with ESS score of >10 (n=47), among them OS patients (n=34)	To evaluate the coexistence of OSA in patients with stable COPD.	• Among COPD patients with excessive daytime sleepiness (ESS score of >10), the coexistence of OSA was observed in 70.2%.<br>• After taking into account the severity of OSA in the OS group, there were 10 patients with mild, 7 with moderate, and 16 with severe OSA.<br>• Patients with OS were older and had higher BMI and neck circumference than COPD-only patients.<br>• OS patients had worse health-related QoL than COPD-only patients.<br>• Age, FEV_1 (% predicted), BMI, neck circumference, evidence of PH, TSH, snoring, and ESS were associated with increased risk of OSA among COPD patients.

Marques 2021 [29]	Cross-sectional study	COPD patients (n=181), among them OS patients (n=116)	To assess the relationship between different domains of lung function and PSG parameters in COPD patients.	• Among COPD patients 64.1% had coexistent OSA, and 82.2% had significant nocturnal desaturation.<br>• FEV_1/FVC was associated with sleep efficiency, whereas DLco predicted sleep onset latency and percentage of REM sleep time /TST.
Ferrer-Lluis 2021 [30]	Retrospective cohort study	COPD patients (n=78), among them OS patients (n=51);<br>control group (n=952), among them OSA patients (n=50)	To evaluate how COPD affects the severity and characteristics of OSA.	• The percentage of non-recovered desaturations in patients with sleep apnea slightly increased with COPD severity.<br>• The median basal SaO_2 value during the night excluding the moments where a desaturation occurred was lower for the COPD patients compared to the control group.<br>• TS90% increased with both the OSA and the COPD severity.
Bae 2021 [31]	Retrospective cohort study	patients with symptoms suggestive of sleep apnea and airway disease who underwent spirometry and PSG (n=355), among them: neither COPD nor moderate/severe OSA patients (n=152), moderate/severe OSA patients without COPD (n=109), COPD patients without moderate/severe OSA (n=57), OS patients (n=37)	To assess mortality in COPD, OSA, and OS, and evaluate what better predicts mortality within 10 years in PSG - AHI or hypoxemic load measurements.	• Both groups of COPD patients with and without moderate/severe OSA had increased all-cause mortality than those who had neither disease, but OS patients had mortality not significantly higher than COPD without moderate/severe OSA patients.<br>• The measurement of hypoxemic load, not AHI, better-predicted mortality within 10 years when compared with the clinical model (not adjusted for data from PSG).
Akinnusi 2021 [32]	Retrospective cohort study	OS patients without any diagnosis of atrial arrhythmias before the date of PSG (n=268)	To evaluate the prevalence of AF among OS patients, to identify risk factors predisposing to AF, and to assess whether AF contributes to the risk of morbidity and mortality.	• Incident AF occurred in 24% of patients.<br>• Independent predictors of incident AF were ACCI, TS90%, and CPAP adherence.<br>• OS patients with AF experienced higher hospitalization rates and worse mortality rates.<br>• ACCI, the severity of airflow obstruction, and CPAP adherence were independent predictors of mortality in OS patients.

Adler 2021 [33]	Prospective cohort study	OS patients (n=754), OSA-only patients (n=5566)	To explore the main differences in the clinical response to CPAP treatment in OS patients compared to patients with OSA alone.	• CPAP efficacy measured on the residual AHI and median adherence were similar between OS and OSA-only patients<br>• The overall burden of symptoms related to OSA improved with CPAP treatment regardless of COPD status.<br>• OS was associated with higher odds of persistent morning headaches, morning tiredness, daytime sleepiness, and exertional dyspnea when compared with OSA alone.
Zhu 2020 [34]	Cross-sectional study	COPD patients (n = 766), among them OS patients (n = 520)	To evaluate the relationship of lung function with the AHI in OS patients.	• After adjustment for age, sex, BMI, neck circumference, economic status, smoking status, alcohol consumption, and hypertension, the FEV_1 has a positive correlation with the AHI in OS patients ($p<0.01$), but there was no correlation with the FEV_1 of each grade and the AHI.<br>• Using multiple regression analyses, the FEV_1 was positively correlated with the nadir SaO_2 ($p<0.01$) and negatively correlated with the TS90% ($p<0.01$) in OS patients.
Zhou 2020 [35]	Cross-sectional study	COPD patients (n=151), among them OS patients (n=28)	To evaluate the metabolic abnormalities and the status of systemic inflammation in OS and COPD-only patients.	• Co-occurrence of OSA was found in 19.2% of COPD patients.<br>• There were significant differences in neck and waist circumference, levels of BP, lipid metabolic, glucose metabolic, and inflammatory biomarkers between OS and COPD-only patients.<br>• MS was more frequent in OS than in COPD-only patients.<br>• BMI, and systolic BP when falling asleep were independent predictors of the presence of MS in OS patients.
Zhang 2020 [36]	Cross-sectional study	COPD patients (n=65), among them OS patients (n=29)	To investigate the impact of OSA on cognitive impairment in COPD patients.	• OS patients had worse results in the MMSE compared to COPD-only patients (mean 25.5 vs. 23.5) and COPD patients with coexistent severe OSA had lower MMSE scores than those with mild OSA (22.6 vs. 25.5).<br>• OS patients had a higher risk to develop dementia than COPD-only patients (66% vs. 31%, based on MMSE score).<br>• Independent risk factors for dementia in COPD patients were older age, lower educational level, and higher ODI.
Yang 2020 [37]	Cross-sectional study	COPD patients (n=124), among them OS patients (n=70)	To evaluate the correlation between OSA and coexisted bronchiectasis in COPD patients.	• 56.45% of COPD patients had coexistent OSA.<br>• Bronchiectasis was detected in 42.86% of OS patients and 18.52% of COPD-only patients.<br>• Compared to COPD with coexisted bronchiectasis patients, in OS with coexisted bronchiectasis patients higher values of CRP, TS90%, and lower CD4/CD8 were detected.

Wu 2020 [38]	Cross-sectional study	COPD patients (n=116), among them OS patients (n=62)	To investigate the performance of BQ, mBQ, and SBQ in screening OSA among COPD patients and investigate how pulmonary function interferes with questionnaire scoring.	• BQ, mBQ, and SBQ scores were suitable for screening OSA among COPD patients, and the accuracy of mBQ was slightly higher than the other two.<br>• In COPD patients, FEV_1% pred and FVC%pred were statistically associated with the risk of OSA misdiagnosis in BQ, mBQ, and SBQ.
Wang 2020 (a) [39]	Cross-sectional study	COPD patients (n=277), among them OS patients (n=106)	To assess the prevalence of MCI in OS and COPD-only patients, and investigate the potential mechanism of OSA impact on the risk of MCI in COPD patients.	• Among COPD patients, 38% had coexistent moderate-to-severe OSA.<br>• OS patients had higher prevalence rates of CHD, DM, and myocardial infarction than COPD-only.<br>• The prevalence of MCI was significantly higher in OS than in COPD-only patients (40.6% vs. 24.6%).<br>• Severe OSA and increased TS90% were associated with increased odds of MCI.
Wang 2020 (b) [40]	Retrospective cohort study	the development group of COPD patients (n=77), the validation group of COPD patients (n=78)	To develop a simplified screening questionnaire to detect the existence of OSA in COPD patients.	• Snoring, witnessed apnea, BMI ≥27.5kg/m2, and CHD were significantly correlated with an AHI ≥30 and were incorporated into the screening questionnaire.<br>• The elaborated questionnaire showed a sensitivity of 85.2-87.5%, a specificity of 69.8-80.4%, a positive predictive value of 56.8-69.7%, and a negative predictive value of 91.1-92.5% for detecting severe OSA.
Hu 2020 [41]	Cross-sectional study	COPD patients (n=968), among them OS patients (n=660)	To assess the prevalence of five comorbidities (hypertension, DM, CVD, arrhythmia, and cerebrovascular disease) in OS and COPD-only patients.	• 68.2% of COPD patients had coexistent OSA.<br>• In OS patients, the frequency of hypertension was higher than in the COPD-only group, whereas the prevalence of DM, CVD, arrhythmia, and cerebrovascular disease did not differ significantly between groups.<br>• The prevalence of hypertension increased with the severity of OSA in OS patients<br>• OSA was an independent risk factor for hypertension.<br>• The prevalence of arrhythmia increased with AFL severity in COPD patients.
Archontogeorgis 2020 [42]	Cross-sectional study	OS patients (n=163)	To evaluate the prevalence and identify predictors of MS in OS patients.	• MS diagnosis was made in 38% of OS patients.<br>• OS patients with and without MS presented similar pulmonary function and sleep parameters.

				• In OS patients with MS, abdominal obesity was the most prevalent component of MS (100%), followed by hypertension (82.3%), hypertriglyceridemia (72.6%), and hyperglycemia (51.6%).<br>• Independent predictors of MS in OS patients were: age (p=0.009) and BMI (p=0.029).
Zhang 2019 [43]	Cross-sectional study	COPD patients (n=73), among them OS patients (n=32);<br>patients with fibrotic ILD (n=77), among them patients with coexistent OSA (n=48)	To identify parameters that predict the occurrence and severity of OSA and to investigate the effect of OSA on QoL in patients with CRDs.	• The prevalence of moderate-to-severe OSA was 44% in COPD and 62% in ILD.<br>• The CAT scores related to sleep quality and daily vitality were worse among patients with than without coexistent OSA.<br>• In patients with CRDs, OSA was associated with the SBQ and ODI from the oximetry recording.<br>• The SBQ with a cutoff point ≥3 or 6 had the highest sensitivity and specificity in detecting OSA in CRDs.<br>• ODI had the best accuracy in identifying OSA and was independently associated with the AHI in CRDs.
Yamaguchi 2019 [44]	Cross-sectional study	OSA patients (n=164), among them OS patients (n=38)	To evaluate PSG features of OS and OSA-only patients focusing on low arousal threshold.	• A low arousal threshold was significantly more frequently exhibited in OS patients with moderate-to-severe AFL than in those with OSA-only and in OS patients with mild AFL.<br>• As FEV_1/FVC decreased in OS patients, the mean length of apnea decreased, hypopnea fractions increased, and AHI decreased.<br>• FEV_1/FVC contributed independently to low arousal threshold in OS patients.
Xiong 2019 [45]	Cross-sectional study	COPD patients (n=431), among them OS patients (n=335)	To compare the performance of the ESS, SACS, BQ, and SBQ in predicting OSA among COPD patients.	• The prevalence of OS among COPD patients was 77.7%.<br>• SBQ performed better in detecting various degrees of OSA in COPD patients when compared with ESS, SACS, and BQ.<br>• For screening mild OSA, the cutoff score of SBQ >2 was considered a high risk of OSA (sensitivity 92,8%, specificity 40.6%, AUC 0.723).<br>• For screening severe OSA, the cutoff score of SBQ was >4 (sensitivity 66.1%, specificity 82.1%, AUC 0.824).
Wang 2019 [46]	Cross-sectional study	OS patients (n=25), COPD-only patients (n=25), OSA-only patients (n=25), HS (n=20)	To assess the prevalence of CV complications in OS patients, and explore if OS patients exhibited vascular endothelial dysfunction and abnormalities in the	• The prevalence of hypertension and CHD was significantly higher in the OS group than in the HS, OSA-only, and COPD-only groups.<br>• The levels of sVCAM-1 and TNFα- were significantly higher in the OS group than in the HS, OSA-only, and COPD-only groups.<br>• The percentage of CD4+ lymphocytes and CD4+/CD8+ were significantly lower in the OS group than in the HS, OSA-only, and COPD-only groups.<br>• There were significant negative correlations in the levels of sVCAM-1 and TNFα with CD4+/CD8+ lymphocytes.

			cellular immune function of T lymphocytes.	
Voulgaris 2019 [47]	Cross-sectional study	patients referred to the sleep laboratory due to symptoms suggestive of sleep-disordered breathing (n=244), among them OS patients (n=42), OSA-only patients (n=139), HS (n=63)	To investigate the 10-year risk for CVD in OS patients compared with OSA-only and HS groups.	• Both FRS and systematic coronary risk evaluation were significantly elevated in the OS group compared with the OSA-only and HS groups.<br>• Age, FEV_1, and ODI were major determinants for the systematic coronary risk evaluation, whereas age and AHI were for the FRS.
Kendzerska 2019 [48]	Prospective cohort study	OS patients (n=10149)	To determine whether the combined presence of COPD and severe OSA defined by the AHI or degree of nocturnal hypoxemia is associated with increased hazards of CV events and mortality.	• At the initial point, 30% of participants had AHI >30, 25% spent at least 10 minutes of sleep with SaO_2 <90%, and 12% had COPD.<br>• The primary outcome was a composite of hospitalization due to myocardial infarction, stroke, congestive heart failure, cardiac revascularization procedures, or death, and was developed by 16.4% of participants over a median of 9.4 years.<br>• A greater hazard of outcome was observed in COPD patients who spent at least 10 minutes of sleep with SaO_2 <90%, but not with AHI >30, and a synergistic effect was found only in women.
Jaoude 2019 [49]	Retrospective cohort study	OS patients (n=225), among them OS patients who had a COPD exacerbation (n=92), OS patients matched on age and BMI who had no COPD exacerbation during the study period (n=92)	To identify clinical modifiable factors associated with COPD exacerbations and all-cause mortality in OS patients.	• There was no significant association between AFL severity and AHI in OS patients (p=0.31).<br>• OS patients who had at least one COPD exacerbation were more likely to be active smokers (p=0.01), have poorer lung function (p=0.001), and are less likely to adhere to CPAP use (0.03).<br>• All-cause mortality was correlated with low FEV_1 (p=0.006), CPAP use (p=0.007), and burden of comorbidities (p=<0.001).
Chang 2019 [50]	Cross-sectional study	COPD patients (n=91)	To evaluate HSAT using the Nox-T3 portable monitor to diagnose OSA in COPD patients.	• HSAT had 95% sensitivity, 78% specificity, 88% positive predictive value, and 89% negative predictive value compared to PSG in diagnosing OSA based on AHI≥5.<br>• A lower proportion of participants met the criteria for moderate-to-severe OSA based on HSAT when compared to results from PSG (P = .012) or in-laboratory portable monitor (p=0.031).<br>• Mean oxygen saturation was 93.2 ± 3.7% on PSG, 91.0 ± 3.3% on

				in-laboratory portable monitor, and 90.8 ± 4.0% on HSAT ($p<0.0001$).<br>• Percentage time $SaO_2 \leq 88\%$ was 17.9 ± 26.4% on HSAT, 17.4 ± 25.5% on the in-laboratory portable monitor, and 10.0 ± 21.1% on PSG ($p<0.0001$).
Alvarez 2019 [51]	Cross-sectional study	patients showing moderate-to-high clinical suspicion of suffering from sleep apnoea (n=297), among them OS patients with moderate-to-severe OSA (n=62), COPD-only patients (n=22), moderate-to-severe OSA-only patients (n=213)	To assess the usefulness of sample entropy to distinguish changes in overnight PRV recordings while sleeping among COPD -only, moderate-to-severe OSA-only, and OS patients with moderate-to-severe OSA.	• Overnight PRV recordings from OS patients were significantly more irregular than those from COPD-only or OSA-only patients.<br>• Sample entropy can properly quantify changes in the overnight CV dynamics of OS patients.
Schreiber 2018 (a) [52]	Retrospective cohort study	OS patients (n=92)	To evaluate the effect of 1 year of CPAP treatment on arterial blood gases and pulmonary function tests in OS patients and its association with the baseline severity of airway obstruction.	• After 1 year of CPAP treatment:<br>- a significant improvement in arterial blood gases in all patients, more pronounced in patients who were hypercapnic at baseline,<br>- no significant change in respiratory function (FEV_1, VC, FEV_1/FVC) in the general population, but a significant improvement in FEV_1 in the subgroup of hypercapnic patients,<br>- patients with a basal FEV_1 below 79.1% significantly improved, whereas patients with a basal FEV_1 above 79.1% significantly worsened.
Schreiber 2018 (b) [53]	Cross-sectional study	COPD patients (n=422), among them OS patients with moderate-to-severe OSA (n=190)	To evaluate the prevalence and predictors of OSA in COPD patients undergoing inpatient pulmonary rehabilitation programs.	• OS patients were significantly younger and had less severe airway obstruction as compared to COPD-only patients.<br>• No significant differences in cardiac comorbidities nor arterial blood gases between OS and COPD-only groups.<br>• OS patients had significantly more severe diurnal symptoms assessed by the ESS and higher BMI compared to COPD-only patients.<br>• 36.3% of OS patients showed an ESS >10.<br>• 25% of OS patients had BMI ≤25.
Pissulin 2018 [54]	Cross-sectional study	COPD patients (n=66), among them OS patients (n=46).	To investigate whether the presence of OSA affects the perception of respiratory	• The confounding factors present in the triad of OSA, COPD, and obesity prevented the perception of increased daytime sleepiness and high risk for OSA.

			symptoms and QoL in COPD patients, and to determine whether scales for assessing daytime sleepiness and for screening for OSA are effective in the triad of OSA, COPD, and obesity.	• There was a significant difference in mean FEV_1 between the OS and the COPD-only group (p = 0.073).<br>• The presence of the triad of OSA, COPD, and obesity did not lead to significantly higher ESS scores, and scores >10 had a specificity of 0.58.<br>• The BQ did not identify a high risk for OSA in the presence of the triad (specificity of 0.31).<br>• There were no significant differences in the domain or total scores of the Saint George's respiratory questionnaire between the OS and the COPD-only group.
Papachatzakis 2018 [55]	Cross-sectional study	OS patients (n=38), OSA-only patients (n=38)	To evaluate the relative prevalence of comorbidities in OS patients compared to OSA-only patients matched for sex, age, and BMI.	• 7.9% of OS patients and 23.7% of OSA-only had no other known coexisting diseases.<br>• OS patients more frequently had at least 4 comorbidities than OSA-only patients (29% vs. 10.5%).<br>• The most common comorbidities in OS and OSA-only groups were hypertension (50% vs. 42.1%), CVD (44.7% vs. 26.3%), DM (29% vs. 13.2%), dyslipidemia (21.1% vs. 26.3%) and depression (7.9% vs. 13.2%).
Economou 2018 [56]	Case-control study	OS patients (n=38), OSA-only patients (n=38), HS (n=28)	To compare ESS, FSS, and HADS between OS and OSA-only patients before and after 3 months of CPAP therapy.	• There was no significant difference in ESS, HADS–Anxiety, and HADS–Depression scores between OS and OSA-only patients.<br>• OS patients complained of significantly more fatigue (FSS) than OSA-only patients, and it persisted after 3 months of CPAP therapy.
Archontogeorgis 2018 [57]	Cross-sectional study	patients referred to the sleep laboratory (n=485), among them OS patients (n=79), OSA-only patients (n=324), HS (n=82)	To evaluate MPV and PDW in OS patients compared to OSA-only and HS groups.	• MPV was significantly lower in HS than in OS and OSA-only groups.<br>• MPV was significantly higher in OS than in OSA-only patients (10.7 vs. 10.3, p=0.002).<br>• PDW was significantly lower in HS than in OS and OSA-only groups.<br>• There was no difference in PDW between OS and OSA groups.

ACCI (age-adjusted Charlson comorbidity index), AF (atrial fibrillation), AFL(airflow limitation), AHI (apnea-hypopnea index), AI (arousal index), AUC (area under the curve), BMI (body mass index), BP (blood pressure), BQ (Berlin questionnaire), CAT (chronic obstructive pulmonary disease assessment test), CCI (Charlson comorbidity index), CHD (coronary heart disease), COPD (chronic obstructive pulmonary disease), CPAP (Continuous Positive Airway Pressure), CRDs (chronic respiratory diseases), CRP (C-reactive protein), CV (cardiovascular), CVD (cardiovascular disease), DLco (diffusing capacity for carbon monoxide), DM – diabetes mellitus, ESS (Epworth sleepiness scale), FEV_1 (the forced expiratory volume in 1 second), FVC (forced vital capacity), FRS (Framingham risk score), FSS (fatigue severity scale), GOLD - Global Initiative for Obstructive Lung Disease, HADS (hospital anxiety and depression scale), Hb (hemoglobin), HS (healthy subjects - in the sense of individuals not suffering from OSA and COPD), HSAT (home sleep apnea testing), ILD (interstitial lung disease), mBQ (modified Berlin questionnaire), MCI (mild cognitive impairment), MMSE (Mini-Mental State Examination), MPV (mean platelet volume), MS (metabolic syndrome), NREM (non-rapid eye movement), ODI (oxygen desaturation index), OS (overlap syndrome of chronic obstructive pulmonary disease and obstructive sleep apnea), OSA (obstructive sleep apnea), $PaCO_2$ (partial pressure of carbon dioxide), PaO_2 (partial pressure of oxygen), PDW (platelet distribution width), PH (pulmonary hypertension), PRV (pulse rate variability), PSG (polysomnography), PWV(pulse wave velocity), REM (rapid eye movement), SACS (sleep apnea clinical score), SaO_2 (oxygen saturation), SBQ (STOP-BANG questionnaire), sVCAM-1 (soluble vascular cell adhesion molecule-1), TNFα (tumor necrosis factor α), TS90% (percentage of total sleep time with oxygen saturation below 90%), TSH (thyroid-stimulating hormone), TST (total sleep time), VC (vital capacity), QoL (quality of life)

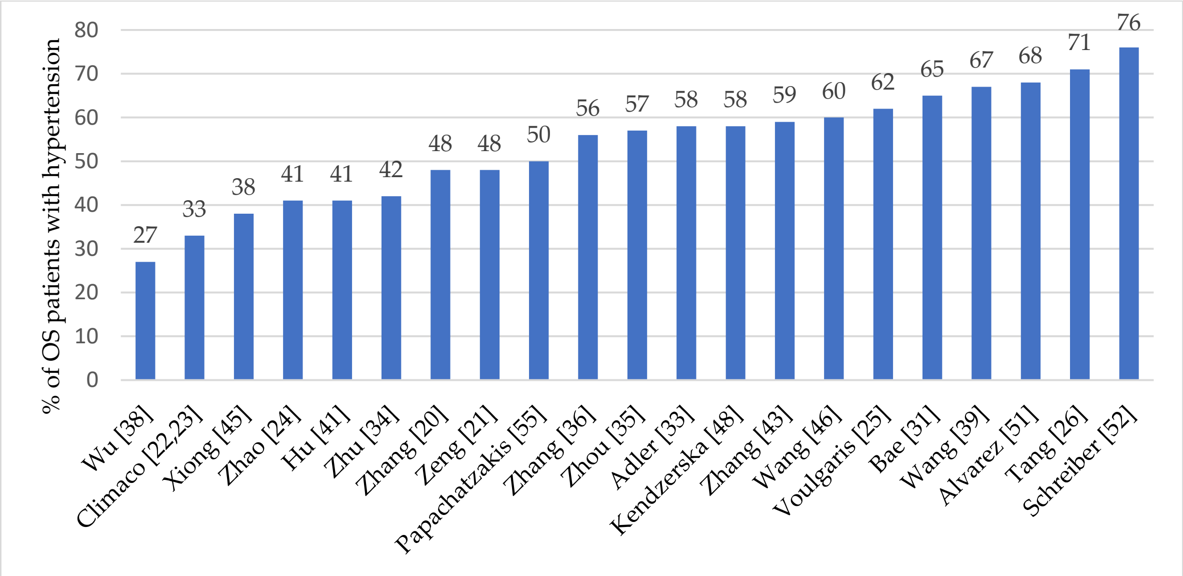

Figure S4. The prevalence of hypertension in OS patients in the included studies.

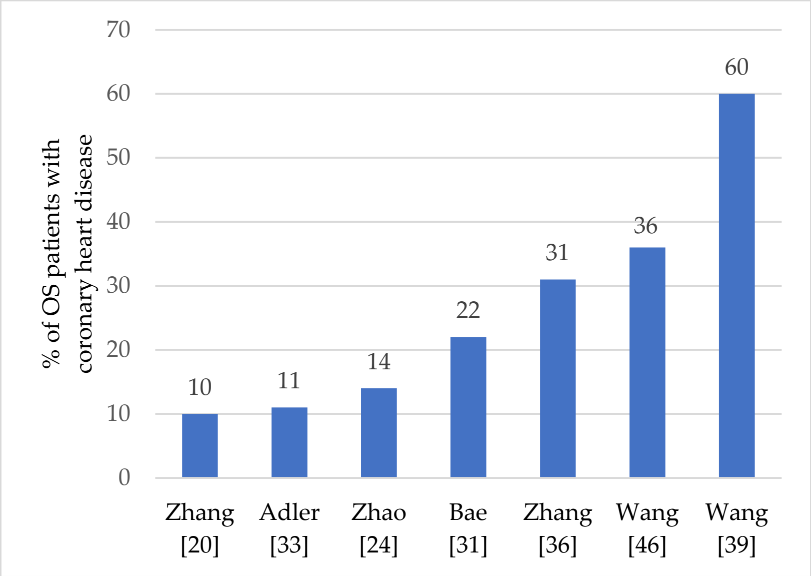

Figure S5. The prevalence of coronary heart disease in OS patients in the included studies.

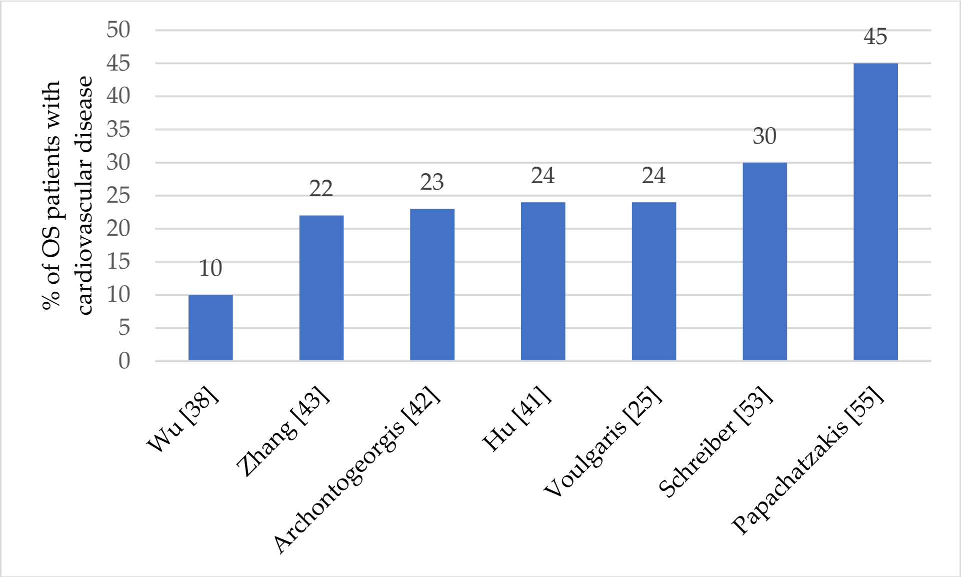

Figure S6. The prevalence of cardiovascular disease in OS patients in the included studies.

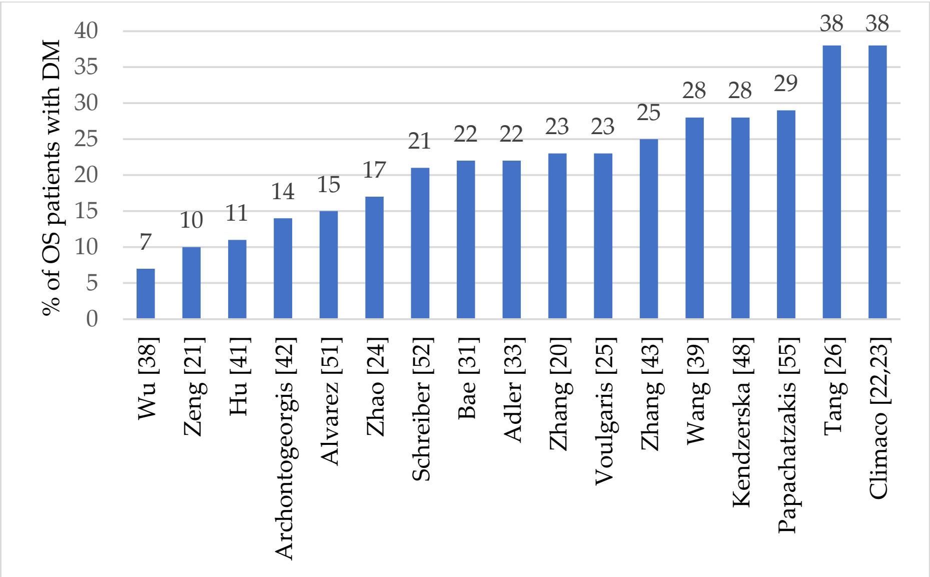

Figure S7. The prevalence of diabetes mellitus in OS patients in the included studies.

References

20. Zhang, P.; Chen, B.; Lou, H.; Zhu, Y.; Chen, P.; Dong, Z.; Zhu, X.; Li, T.; Lou, P. Predictors and outcomes of obstructive sleep apnea in patients with chronic obstructive pulmonary disease in China. *BMC Pulm. Med.* **2022**, *22*, 16. https://doi.org/10.1186/s12890-021-01780-4.
21. Zeng, Z.; Song, Y.; He, X.; Yang, H.; Yue, F.; Xiong, M.; Hu, K. Obstructive Sleep Apnea is Associated with an Increased Prevalence of Polycythemia in Patients with Chronic Obstructive Pulmonary Disease. *Int. J. Chronic Obstr. Pulm. Dis.* **2022**, *17*, 195–204. https://doi.org/10.2147/copd.S338824.
22. Clímaco, D.C.S.; Lustosa, T.C.; Silva, M.; Lins-Filho, O.L.; Rodrigues, V.K.; Oliveira-Neto, L.A.P.; Feitosa, A.D.M.; Queiroga, F.J.P., Jr.; Cabral, M.M.; Pedrosa, R.P. Sleep quality in COPD patients: Correlation with disease severity and health status. *J. Bras. Pneumol.* **2022**, *48*, e20210340. https://doi.org/10.36416/1806-3756/e20210340.
23. Clímaco, D.C.S.; Lustosa, T.C.; de F.P, M.V.; Lins-Filho, O.L.; Rodrigues, V.K.; de Oliveira Neto, L.A.P.; Feitosa, A.D.M.; Queiroga Júnior, F.J.P.; Cabral, M.M.; Pedrosa, R.P. Is obstructive sleep apnea associated with increased arterial stiffness in patients with COPD? *Sleep Breath.* **2022**, 1–6. https://doi.org/10.1007/s11325-022-02635-w.
24. Zhao, Z.; Zhang, D.; Sun, H.; Chang, D.; Lv, X.; Lin, J.; Liu, J.; Wu, X.; Hu, K.; Guo, X.; et al. Anxiety and depression in patients with chronic obstructive pulmonary disease and obstructive sleep apnea: The overlap syndrome. *Sleep Breath.* **2021**, *26*, 1603–1611. https://doi.org/10.1007/s11325-021-02500-2.

25. Voulgaris, A.; Archontogeorgis, K.; Pataka, A.; Flaris, A.N.; Ntolios, P.; Bonsignore, M.R.; Schiza, S.; Steiropoulos, P. Burden of Comorbidities in Patients with OSAS and COPD-OSAS Overlap Syndrome. *Medicina (Kaunas)* **2021**, *57*, 1201. https://doi.org/10.3390/medicina57111201.
26. Tang, M.; Wang, Y.; Wang, M.; Tong, R.; Shi, T. Risk for Cardiovascular Disease and One-Year Mortality in Patients With Chronic Obstructive Pulmonary Disease and Obstructive Sleep Apnea Syndrome Overlap Syndrome. *Front. Pharmacol.* **2021**, *12*, 2882. https://doi.org/10.3389/fphar.2021.767982.
27. Sami, R.; Hashemi, S.; Jalilolghadr, S. Polysomnography findings of patients with overlap syndrome according to severity of lower airway obstruction. *J. Res. Med. Sci.* **2021**, *26*, 130. https://doi.org/10.4103/jrms.JRMS_788_18.
28. Nattusami, L.; Hadda, V.; Khilnani, G.; Madan, K.; Mittal, S.; Tiwari, P.; Mohan, A.; Khan, M.; Guleria, R. Co-existing obstructive sleep apnea among patients with chronic obstructive pulmonary disease. *Lung India* **2021**, *38*, 12–17. https://doi.org/10.4103/lungindia.lungindia-169-20.
29. Marques, R.D.; Berton, D.C.; Domnik, N.J.; Driver, H.; Elbehairy, A.F.; Fitzpatrick, M.; O'donnell, D.E.; Fagondes, S.; Neder, J.A. Sleep quality and architecture in copd: The relationship with lung function abnormalities. *J. Bras. De Pneumol.* **2021**, *47*, e20200612. https://doi.org/10.36416/1806-3756/E20200612.
30. Ferrer-Lluis, I.; Castillo-Escario, Y.; Glos, M.; Fietze, I.; Penzel, T.; Jane, R. Sleep Apnea & Chronic Obstructive Pulmonary Disease: Overlap Syndrome Dynamics in Patients from an Epidemiological Study. In Proceedings of the 2021 43rd Annual International Conference of the IEEE Engineering in Medicine & Biology Society (EMBC), Mexico, 1–5 November 2021; Volume 2021, pp. 5574–5577. https://doi.org/10.1109/embc46164.2021.9630515.
31. Bae, E.; Kwak, N.; Choi, S.M.; Lee, J.; Park, Y.S.; Lee, C.H.; Lee, S.M.; Yoo, C.G.; Cho, J. Mortality prediction in chronic obstructive pulmonary disease and obstructive sleep apnea. *Sleep Med.* **2021**, *87*, 143–150. https://doi.org/10.1016/j.sleep.2021.09.011.
32. Akinnusi, M.; El-Masri, A.R.; Lawson, Y.; El-Solh, A.A. Association of overlap syndrome with incident atrial fibrillation. *Intern Emerg. Med.* **2021**, *16*, 633–642. https://doi.org/10.1007/s11739-020-02469-y.
33. Adler, D.; Bailly, S.; Soccal, P.M.; Janssens, J.P.; Sapène, M.; Grillet, Y.; Stach, B.; Tamisier, R.; Pépin, J.L. Symptomatic response to CPAP in obstructive sleep apnea versus COPD-obstructive sleep apnea overlap syndrome: Insights from a large national registry. *PLoS ONE* **2021**, *16*, e0256230. https://doi.org/10.1371/journal.pone.0256230.
34. Zhu, J.; Zhao, Z.; Nie, Q.; Wang, Y.; Fu, Z.; Guo, X.; Hu, K. Effect of lung function on the apnea-hypopnea index in patients with overlap syndrome: A multicenter cross-sectional study. *Sleep Breath.* **2020**, *24*, 1059–1066. https://doi.org/10.1007/s11325-019-01961-w.
35. Zhou, W.; Li, C.L.; Cao, J.; Feng, J. Metabolic syndrome prevalence in patients with obstructive sleep apnea syndrome and chronic obstructive pulmonary disease: Relationship with systemic inflammation. *Clin. Respir. J.* **2020**, *14*, 1159–1165. https://doi.org/10.1111/crj.13253.
36. Zhang, X.L.; Gao, B.; Han, T.; Xiang, B.Y.; Liu, X. Moderate-to-Severe Obstructive Sleep Apnea and Cognitive Function Impairment in Patients with COPD. *Int. J. Chronic Obstr. Pulm. Dis.* **2020**, *15*, 1813–1822. https://doi.org/10.2147/copd.S257796.
37. Yang, X.; Tang, X.; Cao, Y.; Dong, L.; Wang, Y.; Zhang, J.; Cao, J. The Bronchiectasis in COPD-OSA Overlap Syndrome Patients. *Int. J. Chronic Obstr. Pulm. Dis.* **2020**, *15*, 605–611. https://doi.org/10.2147/copd.S243429.
38. Wu, Q.; Xie, L.; Li, W.; Xiang, G.; Hu, W.; Jiang, H.; Wu, X.; Wu, X.; Li, S. Pulmonary Function Influences the Performance of Berlin Questionnaire, Modified Berlin Questionnaire, and STOP-Bang Score for Screening Obstructive Sleep Apnea in Subjects with Chronic Obstructive Pulmonary Disease. *Int. J. Chronic Obstr. Pulm. Dis.* **2020**, *15*, 1207–1216. https://doi.org/10.2147/copd.S248139.
39. Wang, Y.; Li, B.; Li, P.; Gong, T.; Wu, M.; Fu, J.; Nie, M.; Dong, Y.; Hu, K. Severe obstructive sleep apnea in patients with chronic obstructive pulmonary disease is associated with an increased prevalence of mild cognitive impairment. *Sleep Med.* **2020**, *75*, 522–530. https://doi.org/10.1016/j.sleep.2020.05.002.
40. Wang, T.Y.; Tsai, M.H.; Ni, Y.L.; Lin, T.Y.; Huang, S.Y.; Lo, Y.L. A Simplified Screening Questionnaire for Detecting Severe OSA in Chronic Obstructive Airway Disease in Asian Population. *COPD J. Chronic Obstr. Pulm. Dis.* **2020**, *17*, 191–196. https://doi.org/10.1080/15412555.2020.1733513.
41. Hu, W.; Zhao, Z.; Wu, B.; Shi, Z.; Dong, M.; Xiong, M.; Jiang, Y.; Liu, D.; Li, H.; Hu, K. Obstructive Sleep Apnea Increases the Prevalence of Hypertension in Patients with Chronic Obstructive Disease. *COPD J. Chronic Obstr. Pulm. Dis.* **2020**, *17*, 523–532. https://doi.org/10.1080/15412555.2020.1815688.
42. Archontogeorgis, K.; Voulgaris, A.; Papanas, N.; Nena, E.; Xanthoudaki, M.; Pataka, A.; Schiza, S.; Rizzo, M.; Froudarakis, M.E.; Steiropoulos, P. Metabolic Syndrome in Patients with Coexistent Obstructive Sleep Apnea Syndrome and Chronic Obstructive Pulmonary Disease (Overlap Syndrome). *Metab. Syndr. Relat. Disord.* **2020**, *18*, 296–301. https://doi.org/10.1089/met.2019.0126.
43. Zhang, X.L.; Dai, H.P.; Zhang, H.; Gao, B.; Zhang, L.; Han, T.; Wang, C. Obstructive Sleep Apnea in Patients With Fibrotic Interstitial Lung Disease and COPD. *J. Clin. Sleep Med.* **2019**, *15*, 1807–1815. https://doi.org/10.5664/jcsm.8090.
44. Yamaguchi, Y.; Shiota, S.; Kusunoki, Y.; Hamaya, H.; Ishii, M.; Kodama, Y.; Akishita, M.; Kida, K.; Takahashi, K.; Nagase, T.; et al. Polysomnographic features of low arousal threshold in overlap syndrome involving obstructive sleep apnea and chronic obstructive pulmonary disease. *Sleep Breath.* **2019**, *23*, 1095–1100. https://doi.org/10.1007/s11325-019-01786-7.
45. Xiong, M.; Hu, W.; Dong, M.; Wang, M.; Chen, J.; Xiong, H.; Zhong, M.; Jiang, Y.; Liu, D.; Hu, K. The Screening Value Of ESS, SACS, BQ, And SBQ On Obstructive Sleep Apnea In Patients With Chronic Obstructive Pulmonary Disease. *Int. J. Chronic Obstr. Pulm. Dis.* **2019**, *14*, 2497–2505. https://doi.org/10.2147/copd.S223354.
46. Wang, J.; Li, X.; Hou, W.J.; Dong, L.X.; Cao, J. Endothelial function and T-lymphocyte subsets in patients with overlap syndrome of chronic obstructive pulmonary disease and obstructive sleep apnea. *Chin. Med. J. (Engl.)* **2019**, *132*, 1654–1659. https://doi.org/10.1097/cm9.0000000000000312.

47. Voulgaris, A.; Archontogeorgis, K.; Papanas, N.; Pilitsi, E.; Nena, E.; Xanthoudaki, M.; Mikhailidis, D.P.; Froudarakis, M.E.; Steiropoulos, P. Increased risk for cardiovascular disease in patients with obstructive sleep apnoea syndrome-chronic obstructive pulmonary disease (overlap syndrome). *Clin. Respir. J.* **2019**, *13*, 708–715. https://doi.org/10.1111/crj.13078.
48. Kendzerska, T.; Leung, R.S.; Aaron, S.D.; Ayas, N.; Sandoz, J.S.; Gershon, A.S. Cardiovascular Outcomes and All-Cause Mortality in Patients with Obstructive Sleep Apnea and Chronic Obstructive Pulmonary Disease (Overlap Syndrome). *Ann. Am. Thorac. Soc.* **2019**, *16*, 71–81. https://doi.org/10.1513/AnnalsATS.201802-136OC.
49. Jaoude, P.; El-Solh, A.A. Predictive factors for COPD exacerbations and mortality in patients with overlap syndrome. *Clin. Respir. J.* **2019**, *13*, 643–651. https://doi.org/10.1111/crj.13079.
50. Chang, Y.; Xu, L.; Han, F.; Keenan, B.T.; Kneeland-Szanto, E.; Zhang, R.; Zhang, W.; Yu, Y.; Zuo, Y.; Pack, A.I.; et al. Validation of the Nox-T3 Portable Monitor for Diagnosis of Obstructive Sleep Apnea in Patients With Chronic Obstructive Pulmonary Disease. *J. Clin. Sleep Med.* **2019**, *15*, 587–596. https://doi.org/10.5664/jcsm.7720.
51. Álvarez, D.; Sánchez-Fernández, A.; Andrés-Blanco, A.M.; Gutiérrez-Tobal, G.C.; Vaquerizo-Villar, F.; Barroso-García, V.; Hornero, R.; del Campo, F. Influence of chronic obstructive pulmonary disease and moderate-to-severe sleep apnoea in overnight cardiac autonomic modulation: Time, frequency and non-linear analyses. *Entropy* **2019**, *21*, 381. https://doi.org/10.3390/e21040381.
52. Schreiber, A.; Surbone, S.; Malovini, A.; Mancini, M.; Cemmi, F.; Piaggi, G.; Ceriana, P.; Carlucci, A. The effect of continuous positive airway pressure on pulmonary function may depend on the basal level of forced expiratory volume in 1 second. *J. Thorac. Dis.* **2018**, *10*, 6819–6827. https://doi.org/10.21037/jtd.2018.10.103.
53. Schreiber, A.; Cemmi, F.; Ambrosino, N.; Ceriana, P.; Lastoria, C.; Carlucci, A. Prevalence and Predictors of Obstructive Sleep Apnea in Patients with Chronic Obstructive Pulmonary Disease Undergoing Inpatient Pulmonary Rehabilitation. *COPD J. Chronic Obstr. Pulm. Dis.* **2018**, *15*, 265–270. https://doi.org/10.1080/15412555.2018.1500533.
54. Pissulin, F.D.M.; Pacagnelli, F.L.; Aldá, M.A.; Beneti, R.; Barros, J.L.; Minamoto, S.T.; Weber, S.A.T. The triad of obstructive sleep apnea syndrome, COPD, and obesity: Sensitivity of sleep scales and respiratory questionnaires. *J. Bras. Pneumol.* **2018**, *44*, 202–206. https://doi.org/10.1590/s1806-37562016000000308.
55. Papachatzakis, I.; Velentza, L.; Zarogoulidis, P.; Kallianos, A.; Trakada, G. Comorbidities in coexisting chronic obstructive pulmonary disease and obstructive sleep apnea-overlap syndrome. *Eur. Rev. Med. Pharmacol. Sci.* **2018**, *22*, 4325–4331. https://doi.org/10.26355/eurrev_201807_15429.
56. Economou, N.T.; Ilias, I.; Velentza, L.; Papachatzakis, Y.; Zarogoulidis, P.; Kallianos, A.; Trakada, G. Sleepiness, fatigue, anxiety and depression in Chronic Obstructive Pulmonary Disease and Obstructive Sleep Apnea-Overlap-Syndrome, before and after continuous positive airways pressure therapy. *PLoS ONE* **2018**, *13*, e0197342. https://doi.org/10.1371/journal.pone.0197342.
57. Archontogeorgis, K.; Voulgaris, A.; Papanas, N.; Nena, E.; Froudarakis, M.; Mikhailidis, D.P.; Steiropoulos, P. Mean Platelet Volume and Platelet Distribution Width in Patients With Obstructive Sleep Apnea Syndrome and Concurrent Chronic Obstructive Pulmonary Disease. *Clin. Appl. Thromb. Hemost.* **2018**, *24*, 1216–1222. https://doi.org/10.1177/1076029618788178.
